# Cesium Lead Bromide Perovskites: Synthesis, Stability, and Photoluminescence Quantum Yield Enhancement by Hexadecyltrimethylammonium Bromide Doping

**DOI:** 10.1021/acsomega.2c01490

**Published:** 2022-06-07

**Authors:** Christina Al Tawil, Riham El Kurdi, Digambara Patra

**Affiliations:** Department of Chemistry, American University of Beirut, Beirut 1107 2020, Lebanon

## Abstract

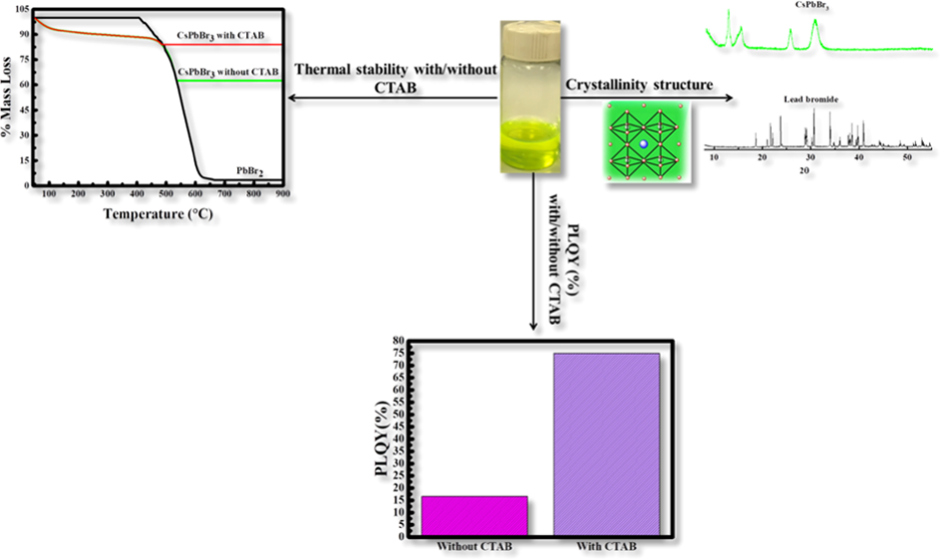

Perovskite nanoparticles having a crystalline structure have attracted scientists’ attention due to their great potential in optoelectronic and scintillation applications. The photoluminescence quantum yield (PLQY) is one of the main critical photophysical properties of the perovskite nanoparticles. Unfortunately, the main limitation of cesium lead halide perovskites is their instability in an ambient atmosphere, where they undergo a rapid chemical decomposition within time. For this purpose, hexadecyltrimethylammonium bromide (CTAB) was used as a surfactant dopant to test in the first place its effect on the stability of CsPbBr_3_ perovskites and on the PLQY values of the prepared perovskites. The addition of CTAB has proven its efficiency in the formed CsPbBr_3_ nanoparticles by increasing their thermal stability and by enhancing their PLQY up to 75%. These results were obtained after the successful preparation of CsPbBr_3_ perovskite nanoparticles by optimizing three different reaction parameters, starting from the time of the reaction, moving to the concentration of lead bromide, and ending with the concentration of cesium oleate. Therefore, it was found that the most stable CsPbBr_3_ perovskites were formed when mixing 0.15 g of lead bromide heated for 40 min with a volume of 1.2 mL of cesium oleate.

## 1 Introduction

The morphology of a perovskite is one of the most important factors that influence the efficiency of the prepared material. For this reason, it is necessary to optimize the synthesis conditions, to achieve the competent product. Indeed, several synthesis methods were developed and improved to produce pure perovskites, such as solid-state reactions,^1^ gas-phase preparation,^2^ wet chemical methods (solution methods),^3^ coprecipitation methods,^4^ hydrothermal synthesis,^5^ sol–gel method,^6^ chemical vapor deposition (CVD) process, and microwave synthesis.^7^ In fact, lead halide perovskite nanowires could be synthesized using the CVD process. This process is based on the vapor–liquid–solid mechanism, where metal films are used as a catalyst to enhance the one-dimensional (1D) crystal growth. One limitation of this method is the low growth temperature of the perovskite. Another synthesis method used is the solution method in the absence of a surfactant ligand. The simple fabrication of perovskite thin films by the solution process results frequently in various uncontrollable defects, such as uncoordinated Pb cations and halide vacancies, inducing the generation of nonradiative recombination sites and the decrease in the photoluminescence quantum yield (PLQY) percentage.^8^

The photoluminescence quantum yield is one of the most essential photophysical properties of perovskites for optoelectronic and scintillation applications. Choosing a luminescent material for use in solid-state lighting is based on the quantum yield. It is defined as the ratio of the number of photons emitted to the number of photons absorbed by an irradiated sample. Henceforward, the optical and electronic properties of lead halide perovskites are dependent on their surface defects. Atomic compositions can vary between the interior and surface of the perovskite crystals. This variation results in an undesirable quantum state within the energy band gap, affecting in a negative way the optoelectronic properties and the photoluminescence stability.^9^ Henceforth, the ligand doping strategy, through the partial substitution of foreign ions for native ions, has gradually become an effective method for significantly enhancing the comprehensive properties of CsPbBr_3_. Even biomolecules, such as DNA and peptide molecules, could serve as an exceptional ligand in the synthesis of inorganic perovskites.^10^

Increased research into the addition of a stabilizing surfactant to perovskites was shown to improve the quantum yield values.^11^ For instance, increasing the surface passivation via salt solutions,^12^ generating other cation/anion compositions through doping,^13^ or postsynthetic ion exchange^14^ are widely investigated. Many researchers have elaborated the method of chemical passivation to minimize the defect formation, to overcome this issue. Moreover, the ligand exchange at the perovskites’ surface stabilizes the perovskite and enhances its PLQY. Therefore, different ligands were used in the literature, where the PLQY percentages obtained were equal to 64% with bismuth doping,^15^ 69% for methyl ammonium addition,^16^ 94% for amino acid (Trp)-mediated synthesis,^10^ and a maximum of 59% for hexadecyltrimethylammonium bromide (CTAB) doping.^17^

Despite the synthesis method adopted, controlling the process parameters such as concentration of precursors (Cs:Pb ratio),^18^ reaction time, and addition of solvents plays an essential role in the perovskites’ quality, crystallinity, crystal size, conversion of the precursor to the perovskite, and efficiency. The formation of the perovskite depends strongly on the precursor’s concentration, temperature, environment, etc.^19,20^ Thus, it is primarily essential to find the optimum route while synthesizing a perovskite, to acquire the best efficiency.

Consequently, in this work, the synthesis of cesium lead halide perovskites was carried out based on the hot injection method using a surfactant ligand to control the synthesis of inorganic perovskite CsPbX_3_ nanoparticles. Furthermore, the addition of CTAB was established to increase the stability and the PLQY of the prepared CsPbBr_3_ perovskites.

## 2 Materials and Methods

### 2.1 Materials

Cesium carbonate (Cs_2_CO_3_), 1-octadecene (ODE, C_18_H_36_), oleylamine (OAm, C_18_H_37_N), hexadecyltrimethylammonium bromide (CTAB, C_19_H_42_BrN), and sulfuric acid (H_2_SO_4_) were obtained from Acros Organics. Lead(II) bromide (PbBr_2_) was acquired from Fisher Scientific company. Oleic acid (OA, C_18_H_34_O_2_) and hexane (C_6_H_14_) were purchased from Sigma Aldrich. Quinine anhydrous (C_20_H_24_N_2_O_2_) was acquired from Fluka Analytical.

All chemicals were used as received without further purification.

### 2.2 Synthesis of Cesium Oleate Solution (Cs-oleate)

Cs-oleate was prepared by mixing 0.4 g of Cs_2_CO_3_ (*C* = 0.053 M) with 20 mL of ODE and 1.24 mL of OA into a vial. The solution was stirred and heated at 200 °C until complete dissolution of cesium carbonate. After complete dissolution, the solution color turned from transparent to yellow, verifying the formation of cesium oleate. Cs-oleate solution was sealed and stored at room temperature for further use.

### 2.3 Synthesis of Hexadecyltrimethylammonium Bromide Solution (CTAB Solution)

The CTAB solution was prepared by mixing 0.05 g with 10 mL of ODE and 0.62 mL of OA into a vial. The solution was stirred and heated at 200 °C until complete dissolution of CTAB, and the color turned from transparent to yellow. The resultant solution was sealed and stored at room temperature for further use.

### 2.4 Synthesis of Cesium Lead Bromide Perovskites

**Figure 1:**
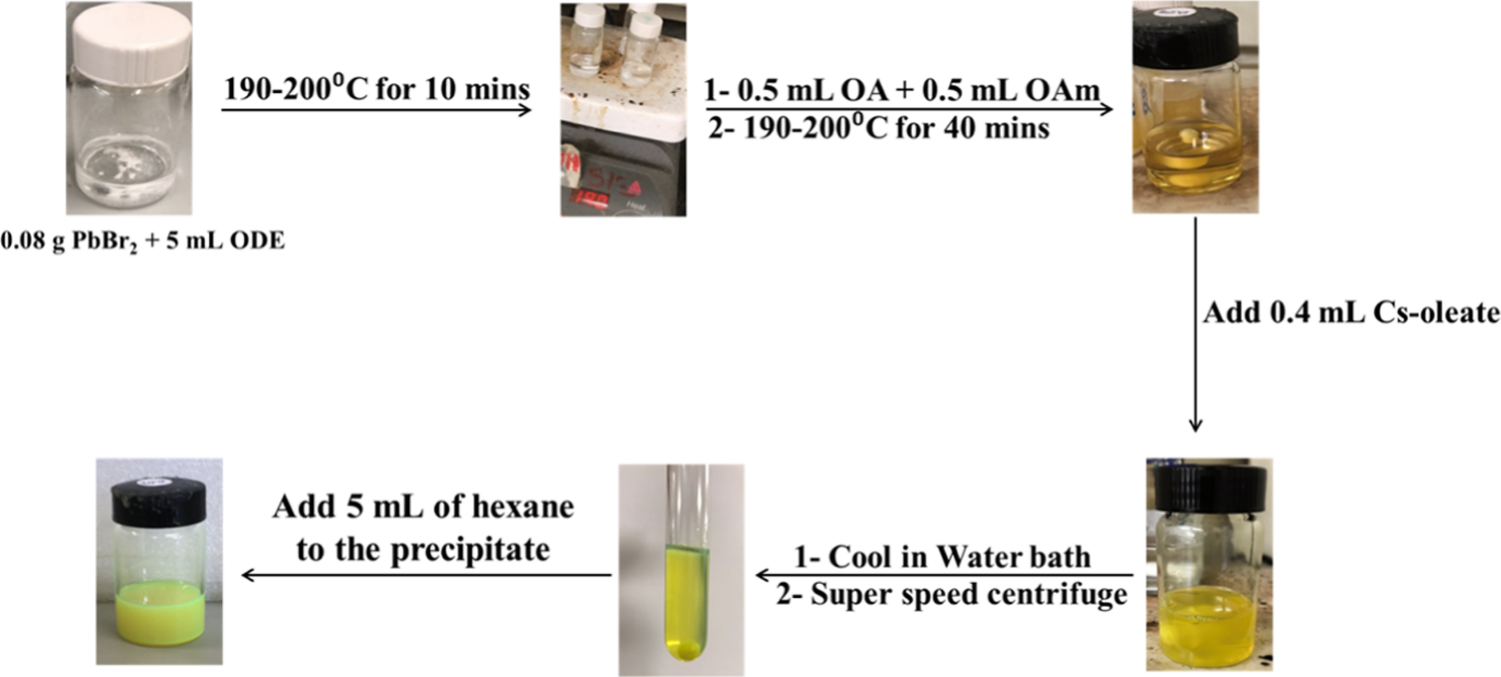
Schematic representation of the CsPbBr_3_ synthesis.

The synthesis of cesium lead halide perovskites was done based by the hot injection method with some modifications.^18^ In the first place, 0.08 g of lead bromide (*C* = 0.0363 M) were mixed with 5 mL of ODE in an air-free environment at 190–200 °C for 10 min. Then, 0.5 mL of OA and 0.5 mL of OAm were added, and the mixture was left until complete dissolution of PbBr_2_ in solution. Next, 0.4 mL of Cs-oleate was added to the solution and immediately immersed in a cold-water bath (*T* = 10 °C), to ensure the formation of the perovskites. Finally, the solution was centrifuged at 15 000 rpm for 15 min, and the precipitate was dissolved in 5 mL of hexane and used for further characterization (See Figure 1).

### 2.5 Optimization of the CsPbBr3 Reaction Parameters

Essentially, the physical properties of the perovskites such as the size and the shape depend intensely on the reaction parameters. For this reason, the reaction parameters were optimized to prepare the most stable lead bromide perovskites. For this purpose, different reaction times were studied, the concentration of lead bromide was established, and finally, the concentration of cesium oleate was also investigated.

#### 2.5.1 Effect of the Reaction Time

In this step, four different solutions were prepared. Each solution contained 0.08 g of lead bromide and was prepared using the same preparation method mentioned in Section 2.3. Hence, in this part, the addition of cesium oleate was done at four different times; once the lead bromide was dissolved (*t* = 0 min), after 10 min; and 20 and 40 min of dissolving. After that, the solutions were centrifuged, and the precipitate was dissolved in hexane for further characterization.

#### 2.5.2 Effect of the Cesium Oleate Concentration

After choosing an adequate reaction time, the effect of the cesium oleate concentration was elaborated. Consequently, four solutions were prepared by dissolving 0.08 g of lead bromide in 5 mL of octadecene, and the synthesis was continued as described in Section 2.3. Then, different volumes of cesium oleate (0.4, 0.8, 1.2, and 2 mL) were added to the mixture of lead bromide. Thus, four solutions were prepared having different concentrations equal to 0.0034, 0.0065, 0.0092, and 0.0138 M. After that, the solutions were centrifuged; the precipitate was dissolved in hexane and then characterized.

#### 2.5.3 Effect of the Lead Bromide Concentration

The final effect established was the effect of the lead bromide concentration. Accordingly, four solutions were prepared by dissolving different masses of lead bromide (0.04, 0.08, 0.15, and 0.2 g) in 5 mL of ODE, and the synthesis was continued as described in Section 2.3. Hence, four solutions were prepared having different concentrations equal to 0.01816, 0.0363, 0.06812, and 0.09082 M. Subsequently, the solutions were centrifuged; the precipitate was dissolved in hexane and finally characterized.

### 2.6 Preparation of Cesium Lead Bromide Perovskites in the Presence of CTAB

Indeed, the effect of CTAB was expanded, after the successful preparation of CsPbBr_3_. For this reason, one solution was prepared as mentioned in Section 2.4 until the complete dissolving of PbBr_2_ in solution. Later on, 2 mL of CTAB solution was added followed by the addition of 0.4 mL of Cs-oleate. The solution was immediately immersed in a cold-water bath (*T* = 10 °C) and centrifuged. The precipitate was dissolved in 5 mL of hexane for additional analysis.

### 2.7 Sample Preparation for PLQY Measurements

In the first step, 1 mM quinine solution was prepared by weighing 1.6 mg of quinine and dissolving it with 5 mL of 0.5 M H_2_SO_4_ solution. In the second step, new samples of cesium lead bromide perovskites were prepared at four different timings. The fluorescence emission intensity of the synthesized CsPbBr_3_ and quinine alone was measured. The PLQY measurements were done at 200 and 40 °C.

### 2.8 Characterization and Spectroscopic Analysis

Initially, the optical properties of the formed perovskites were studied using ultraviolet–visible (UV–vis) spectroscopy. A JASCO V-570 UV–vis–NIR spectrophotometer was used to record the absorption spectra at room temperature in a wavelength range of 400–550 nm in a 3 mL cuvette. Fluorescence emission spectra were measured using a Jobin-Yvon-Horiba Fluorolog III fluorometer and the FluorEssence program. The excitation source was a 100 W Xenon lamp, and the detector used was an R-928 instrument. The excitation and emission slit widths were kept at 5 nm. Briefly, for both measurements, 0.2 mL from the initial solution was pipetted and diluted with hexane into a total volume of 3 mL.

Additionally, the structural properties of the perovskites were examined using X-ray diffraction (XRD). These data were recorded using a Bruker d8 Discover X-ray diffractometer equipped with Cu Kα radiation (λ = 1.5405°). The monochromator used was Johansson-type. The X-ray scans were done for 2θ between 5° and 55°. The step size was 0.02 s, and the scan rate was 20 s per step. For the X-ray study, few drops of the CsPbBr_3_ were deposited on a zero-background holder, and the analysis was conducted. Furthermore, the thermogravimetric analysis (TGA) measurements were done using a Netzsch TGA 209 in the temperature range of 30–900 °C with an increase of 15 °C/min in a N_2_ atmosphere. For this purpose, few μL was measured in an Al_2_O_3_ crucible and then placed in an oven at 45 °C to evaporate the hexane. The complete evaporation of hexane was verified by the consistency of the crucible mass after 20 min. In addition, the surface morphologies were determined scanning electron microscopy (SEM). This analysis was done using a Tescan Vega 3 LMU with an Oxford EDX detector (Inca XmaW20). In short, the lead perovskite solution was deposited on an aluminum stub and coated with carbon conductive adhesive tape.

## 3 Results and Discussion

To begin with, three essential solvents were used. Initially, oleylamine was used as a capping ligand of Pb^2+^, which reduces its reactivity. It strongly coordinates to the Pb ions and binds to the different facets of CsPbBr_3_, which leads to an anisotropic and one-dimensional crystal growth, producing perovskite nanoparticles. Henceforward, oleic acid was used as a surfactant playing the role of a protection ligand along with OAm. It also enhances the growth rate of the nanocrystals and controls its size. Finally, octadecene was considered as a noncoordinating solvent; it is the precipitation medium that induces the perovskite lattice formation. Therefore, it is a good manner to evaluate the effect of the reaction time and the concentration of both cesium oleate and lead bromide.

### 3.1 Optimization of the Reaction Parameters

The preparation of CsPbBr_3_ perovskites was carried out through one simple synthesis route by the hot injection method. Hence, many shapes and sizes could be obtained when varying the reaction parameters. Therefore, different modifications were established, and the obtained perovskites were compared and characterized through XRD, TGA, SEM, UV–vis, and fluorescence spectroscopy.

#### 3.1.1 Reaction Time

**Figure 2:**
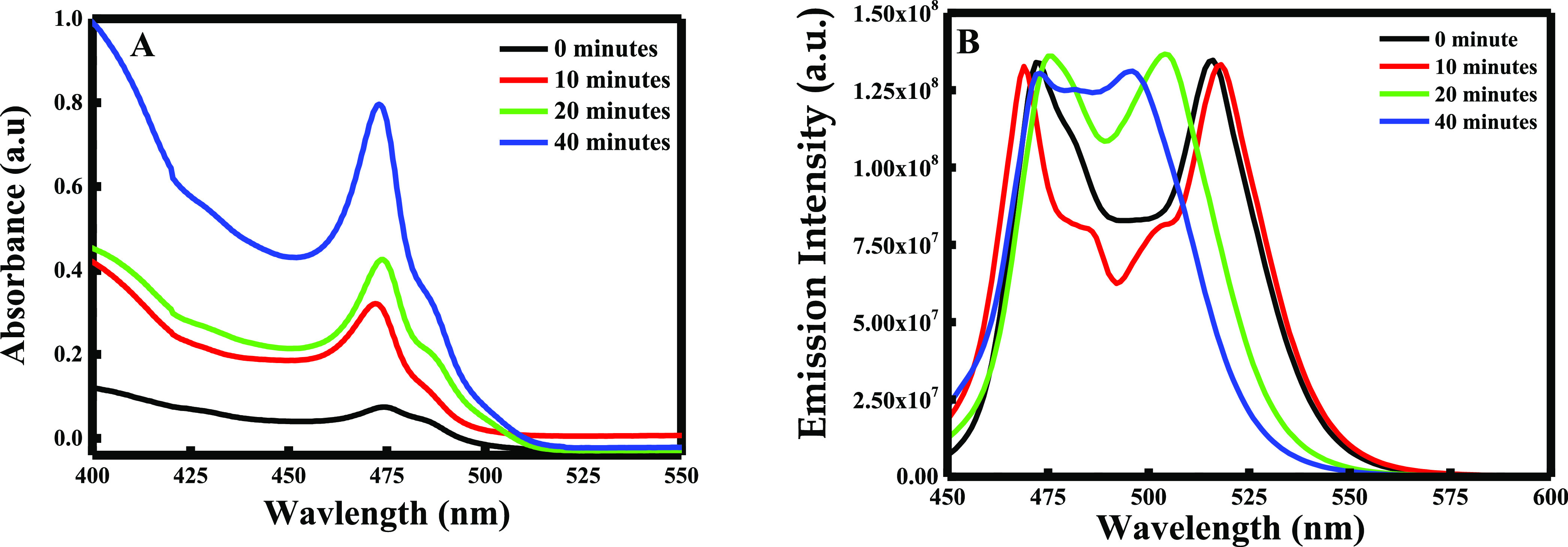
(A) UV–visible spectra and (B) fluorescence emission spectra of lead bromide perovskites having different reaction times.

Four different solutions of lead bromide were prepared. Once lead bromide was dissolved, cesium oelate was added at four different times: *t* = 0, 10, 20, and 40 min. According to De Gruijter et al., PbBr_2_ precursor crystals showed a peak at 330 nm (*S*_0_ → *P*_1_ transition).^21^ However, the absorption of PbBr_2_ perovskites appears at ∼475 nm as shown in Figure 2A. Thus, the identification of absorption ∼475 nm makes it easier for establishing the formation of the perovskites in the solution. The absorption peak at 475 nm remained constant with the increase in the reaction time. These results were similar to the ones established by Belarbi et al. where it was found that perovskites prepared from PbBr_2_ show no significant shift over different reaction times.^22^ Thus, the main difference between the reaction times was observed in the absorbance value. It is remarkable that the absorbance increases as the reaction time increases. Hence, the highest absorbance was obtained when cesium oleate was added after 40 min, meaning that the formation of lead bromide perovskites is higher when the reaction mixture is heated for a longer time. This can be due to the fact that with time, maximum quantities of lead bromide molecules are being combined to OAm and OA, inducing the enhancement of the CsPbBr_3_ yield.

Moreover, the successful production of perovskites was verified through the fluorescence emission spectra. The maximum emission peak of lead bromide perovskites was proved to be around 500 nm.^20^ Changing the reaction times showed a variation in the emission maximum position, and a blue shift of the emission peak was reported with the significant decrease in the crystal size.^22^ These changes are very clear in Figure 2B. In fact, for a different reaction time, two distinctive peaks were obtained (see Table SI.1), where a blue shift appeared with time. In fact, the presence of two separate peaks reveals the presence of two different components in solution. Hence, according to Fang et al., the peak obtained at a lower wavelength is attributed to the pure PbBr_2_; thus, the peak obtained at a higher wavelength is attributed to the pure CsPbBr_3._^23^ Actually, for the different times, the peak at 472 nm remains constant, verifying its attribution to the pure PbBr_2_. It is clear that after 40 min, the two peaks are being additive to form one broad peak. This change in the peak’s shape verifies the complete reaction of PbBr_2_ in solution with cesium oleate to form fine and small CsPbBr_3_ NPs. Hence, the blue shift of the emission intensity wavelength is related to the NP size. In other words, as a blue shift occurred, smallest NPs are formed. This statement was proven by SEM analysis. Actually, clear differences in the crystal morphology were observed. As shown in Figure SI.1A–D, when the reaction time increases, the particle size decreases. These results indicate that 40 min is needed to have a complete reaction and to synthesize the smallest perovskites. However, for *t* = 0 and 10 min, large NPs were formed *(s*ee Figure SI.1A and B*)*. Thus, when reaching 20 min, the CsPbBr_3_ starts to be formed in smaller size and tends to be more uniform (see Figure SI.1C). However, at 40 min, small and uniform CsPbBr_3_ nanoparticles were obtained (see Figure SI.1D), confirming the peak shapes obtained in the fluorescence emission intensity analysis.

#### 3.1.2 Concentration of Cesium Oleate

**Figure 3:**
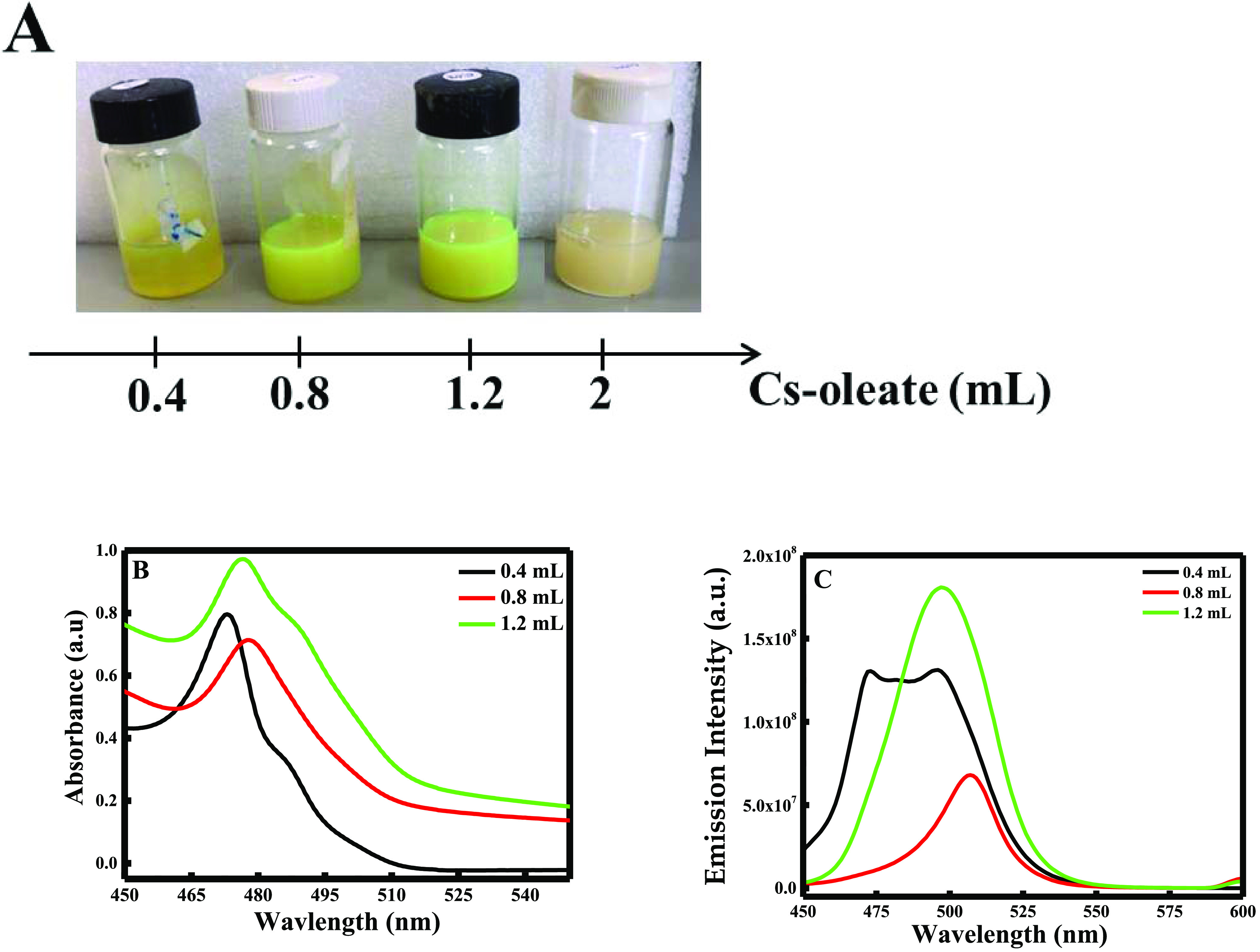
(A) Color change of CsPbBr_3_ solution with the increase of Cs-oleate concentration, (B) UV–visible spectra, and (C) fluorescence emission spectra of lead bromide perovskites after adding different volumes of cesium oleate.

In this part, four different solutions of lead bromide were prepared to add different volumes of Cs-oleate (0.4, 0.8, 1.2, and 2 mL). Hence, Cs-oleate was added after 40 min to ensure that all the PbBr_2_ was reacted with OAm and OA. It is important to mention that when adding 2 mL of cesium oleate, the reaction did not occur, and a pale white-yellow color appeared after centrifugation, meaning that cesium oleate is in excess and inhibited the formation of CsPbBr_3_ (see Figure 3A). Thus, the comparison was done for the three remaining experiments having concentrations equal to 0.0034 M (*V* = 0.4 mL), 0.0065 M (*V* = 0.8 mL), and 0.0092 M (*V* = 1.2 mL). As reported in the literature, the absorption in the visible region is characteristic of the CsPbBr_3_ with an absorption edge at a wavelength below 550 nm.^24^ According to the results obtained in Figure 3B, as the volume of cesium oleate increases, a slight red shift is obtained from 473 to 477 nm. Hence, this minimal shift has no remarkable effect of the size of the NPs. As a consequence, the absorbance value increases, as we increase the volume of cesium oleate, meaning that the reaction of PbBr_2_ with Cs-oleate increases proportionally, inducing therefore the formation of a higher amount of CsPbBr_3_ NPs. These results were in accordance with the results obtained with Shi et al.^25^

Remarkably, as shown in Figure 3C, when adding 0.8 mL of Cs-oleate, a sharp peak was obtained at 495 nm. Similarly, the same peak was obtained when adding 1.2 mL of Cs-oleate with higher emission intensity, around ∼4-fold enhancements. Thus, a broad peak was obtained when adding a small quantity of Cs-oleate (0.4 mL). These results indicate that an excess amount of Cs-oleate (1.2 mL) is needed to ensure the complete reaction of PbBr_2_ with Cs-oleate to produce pure CsPbBr_3_ NPs. It is important to mention that the decrease in the emission intensity when adding 0.8 mL compared to the emission intensity when adding 0.4 mL of cesium oleate is due to the complete absence of PbBr_2_ in the solution. Furthermore, SEM analysis was conducted to confirm the difference between the three different NPs. As shown in Figure SI.2A–C, the increase in the Cs-oleate volume encourages the formation of small, fine, and uniform NCs. Hence, the particle size decreases from 70–80 nm when adding 0.4 and 0.8 mL of Cs-oleate (see Figure SI.2A and B) to 10–20 nm when adding 1.2 mL of Cs-oleate (see Figure SI.2C).

#### 3.1.3 Concentration of Lead Bromide

To investigate the effect of lead bromide concentration, 4 different solutions were prepared by varying the mass of PbBr_2_ from 0.04 to 0.08 g, 0.15, and 0.2 g. After 40 min, 1.2 mL of cesium oleate was added, and the final solution was centrifuged at 15 000 rpm for 15 min.

**Figure 4:**
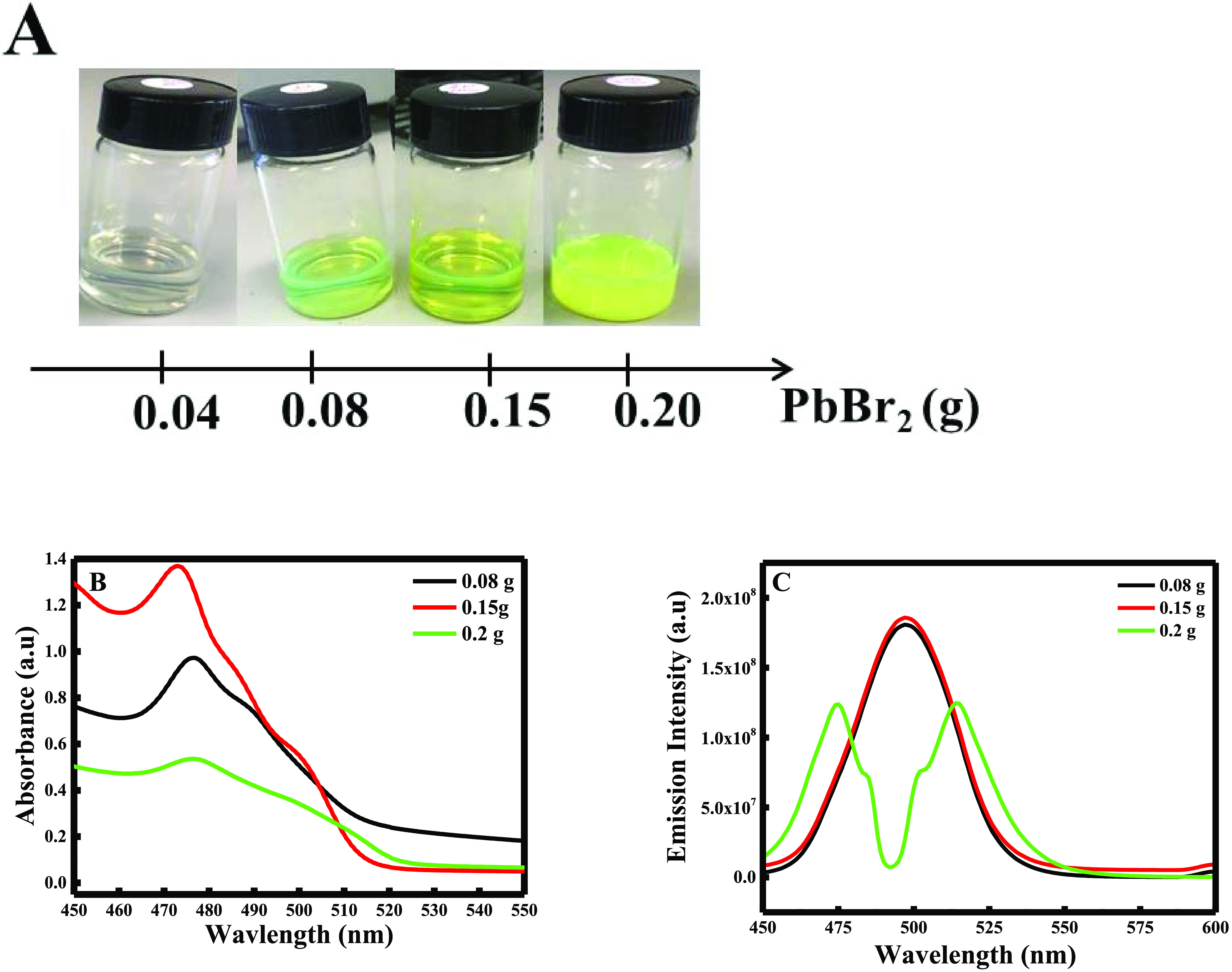
(A) Color change of the CsPbBr_3_ solution with the increase of PbBr_2_ mass; (B) UV–visible spectra; and (C) fluorescence emission spectra of lead bromide perovskites using different masses of lead bromide.

Interestingly, when adding 0.04 g of lead bromide, the reaction did not occur, where after centrifugation, no precipitate was formed (see Figure 4A). Thus, the comparison and the characterization were done for the remaining experiments (0.08, 0.15, and 0.2 g) having concentrations equal to 0.0363, 0.06812, and 0.09082 M, respectively.

Generally, the enhancement of the lead precursor concentration boosts the formation of CsPbBr_3_ NPs. Hence, when increasing the mass of PbBr_2_ from 0.08 to 0.15 g, the absorbance increases proportionally, meaning that cesium lead bromide perovskites are being formed, and the yield is increasing with enhanced crystallization (see Figure 4B).

However, the absorbance decreases again when adding 0.2 g of lead bromide with a broadening peak. This change in the peak is due to the fact that lead bromide is present in excess in the solution with CsPbBr_3_. These results were proven when measuring the emission intensity, where two distinctive peaks were obtained when 0.2 g of PbBr_2_ was added (see Figure 4C).

**Figure 5:**
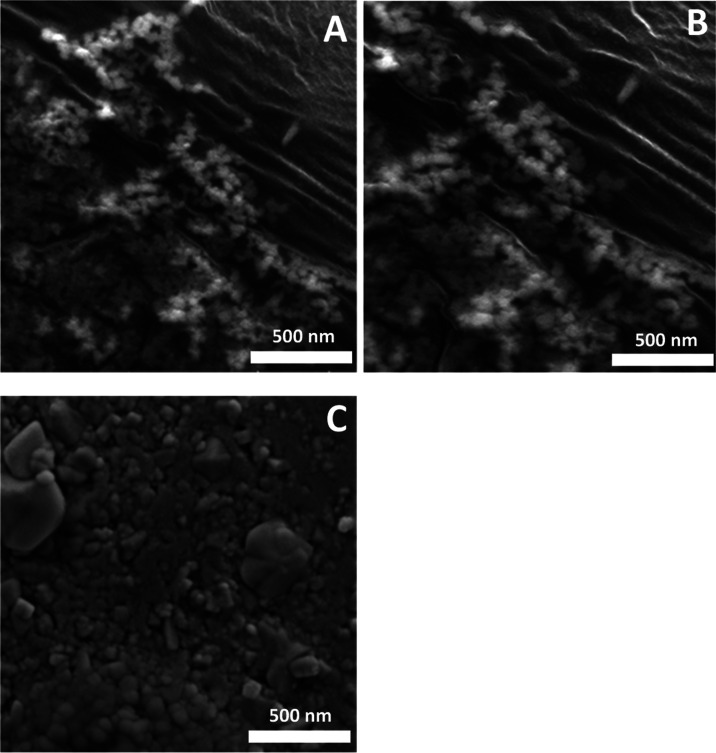
SEM images of lead bromide perovskites having different masses of lead bromide, (A) 0.08 g; (B) 0.15 g; and (C) 0.2 g, in the presence of 1.2 mL of cesium oleate.

Hence, the emission intensity of both CsPbBr_3_ when adding 0.08 and 0.15 g was slightly enhanced, proving that 0.15 g is enough to have a maximum yield of CsPbBr_3_ NPs. Finally, the SEM images are presented in Figure 5A–C, where the NPs increase in size when adding 0.2 g of PbBr_2_ and remain the same when adding 0.08 and 0.15 g of the lead precursor.

### 3.2 Crystallinity Analysis of Lead Bromide Perovskites

To sum up, the best CsPbBr_3_ NPs were obtained after 40 min when mixing 0.15 g of PbBr_2_ with 1.2 mL of Cs-oleate. To further establish the physical properties of the synthesized perovskites, lead bromide and the synthesized CsPbBr_3_ were analyzed using the X-Ray diffraction (XRD) technique. The diffractograms are illustrated in Figure SI.3. The main characteristic peaks of lead bromide appearing at diffraction angles of 2Θ° equalled to 17.49, 22.919, 24.833, 26.111, 27.82, 34.045, 35.405, 39.041, 39.75, 40.602, 42.597, and 43.075°.^26^ However, as it is shown in the diffractogram of the synthesized nanoparticles, these peaks were completely absent. Hence, this confirms the formation of CsPbBr_3_ perovskites. Moreover, the XRD pattern of CsPbBr_3_ was studied by Boote et al.,^27^ where the results showed that CsPbBr_3_ presents several diffraction peaks at 2θ equal to 15, 15.2, 30.4, and 30.7°. Similar results were obtained for the CsPbBr_3_ prepared under our conditions. In addition, three-dimensional (3D) CsPbBr_3_ and two-dimensional (2D) CsPb_2_Br_5_ structures were studied by Acharyya et al.^28^ The obtained XRD diffractograms proved that if the solution is heated continuously after the addition of cesium oleate, the 3D structure of CsPbBr_3_ will be relaxed and transformed into a 2D structure. However, if the solution is directly quenched after the addition of cesium oleate, the 3D structure will be maintained. Hence, the formed CsPbBr_3_ was present in the 3D structure, similar to the results obtained by Acharyya et al.^28^

### 3.3 Photoluminescence Stability of CsPbBr_3_ in the Presence and Absence of CTAB

**Figure 6:**
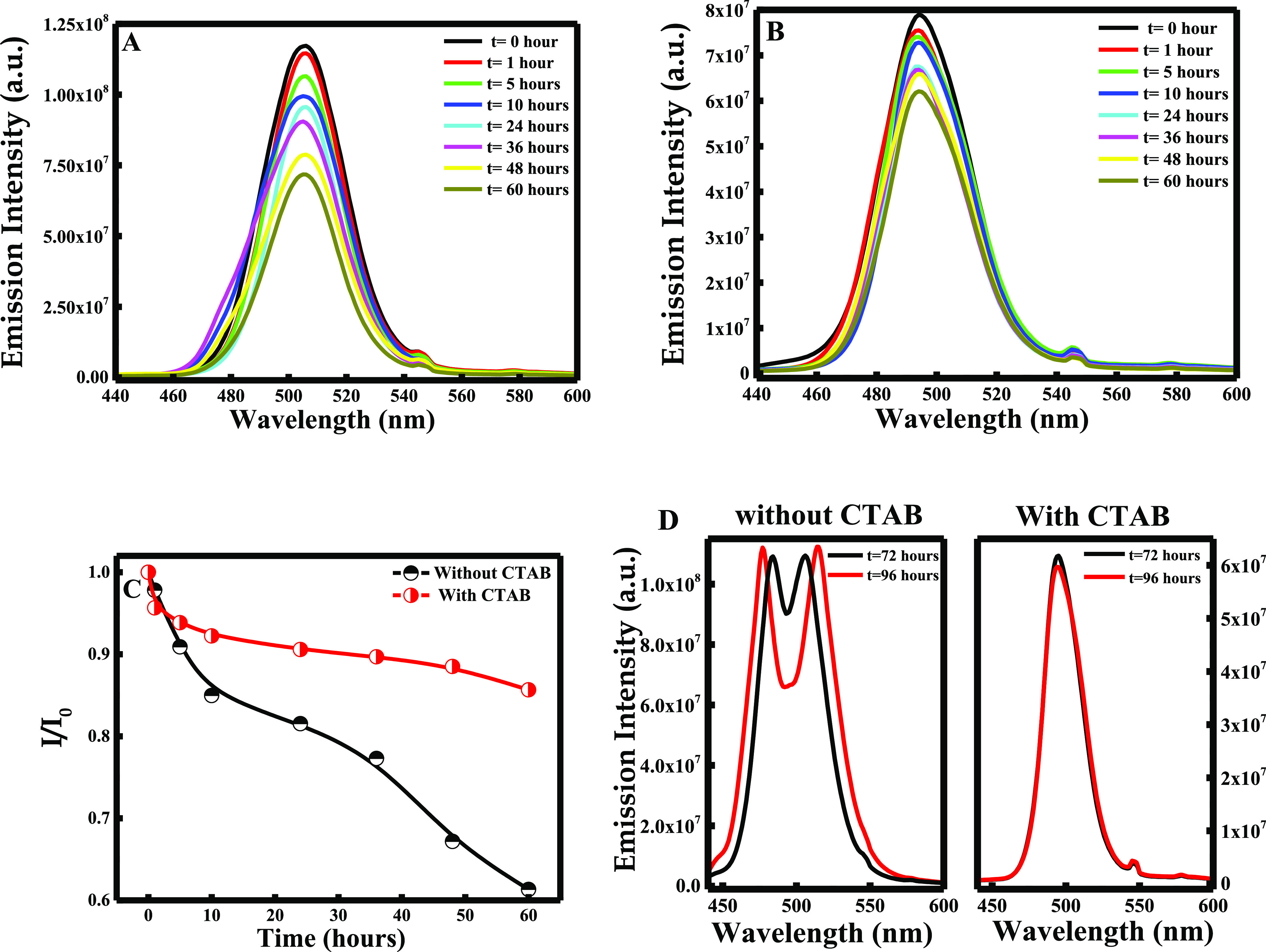
Fluorescence emission intensity of CsPbBr_3_ (A) in the absence of CTAB and (B) in the presence of CTAB; (C) plot of *I*/*I*_0_ for CsPbBr_3_ in the absence and presence of CTAB within time from 0 to 60 h; and (D) difference in the shape of PL intensity peaks in the absence and the presence of CTAB after 60 h.

The fluorescence emission spectrum of the prepared solutions was measured for different times to access the photoluminescence (PL) stability of the perovskites in the absence and presence of CTAB. As shown in Figure 6A,B, the emission intensity of CsPbBr_3_ decreased gradually within time in the presence and absence of CTAB. However, after 24 h, the PL intensity of the prepared CsPbBr_3_ in the presence of CTAB remained almost constant, whereas the PL intensity for the NPs prepared without CTAB decreased consistently. The difference in the PL intensity in the absence and presence of CTAB is remarkable when plotting *I*/*I*_0_ vs time as depicted in Figure 6C.

A similar pattern was observed according to Liu et al, where the CsPbBr_3_ perovskites lost 62% of their PL intensity after 4 days.^29^ Obviously, the decrease was rapid for the solution lacking CTAB, where the PL intensity decreases by around ∼40%. However, this loss was slower for the solution containing CTAB where the PL intensity of CsPbBr_3_ decreased only by ∼15% after the same period of time. Remarkably, as shown in Figure 6D, after 60 h, the PL intensity of CsPbBr_3_ increases, complemented with a peak split into two distinctive peaks. In fact, the presence of two separate peaks reveals the presence of two different components in the solution. As proved earlier, according to Fang et al., the peak obtained at a lower wavelength is attributed to the pure PbBr_2_.^23^^23^ This proves that CsPbBr_3_ perovskites were degraded and the PbBr_2_ precursor was free again in the solution. Interestingly, in the presence of CTAB, the shape of the PL intensity peaks remains unchanged (see Figure 6D). Thus, the incorporation of PbBr_2_ inside the perovskites was maintained by enhancing their stability in the presence of CTAB.

### 3.4 Thermal Stability of CsPbBr_3_ in the Presence and Absence of CTAB

Thermogravimetric analysis was performed to assess the stability of the prepared nanoparticles. As shown in Figure SI.4, PbBr_2_ totally loses its mass upon reaching a temperature slightly below 700°C. However, no mass loss occurred between 100 and 400 °C. However, for both CsPbBr_3_ prepared in the absence and in the presence of CTAB, almost 10% of their mass was lost. Hence, this mass loss is initially related to the presence of hexane. Henceforth, the weight loss of CsPbBr_3_ in both cases occurred at 450 °C, similar to pure PbBr_2_. Thus, CsPbBr_3_ prepared without CTAB loses around 40% of its mass. In fact, this weight loss was proven to be due to the removal of the capped alkyl amines at low temperatures and the sublimation of PbBr_2_ at high temperatures.^28^ These results were in accordance with the TGA analysis done by Xu et al.^30^ Thus, the difference in the degradation pattern of the two components in the absence and in the presence of CTAB means that PbBr_2_ inside the perovskite is less degraded and is thus more stable. However, the addition of CTAB decreases the degradation of the CsPbBr_3_ and decreases its decomposition, where it loses only 15% of its total mass. Hence, the increase in the thermal stability in the presence of CTAB is due to the fact that CTAB molecules contain methylammonium groups, enhancing therefore the incorporation of PbBr_2_ inside the perovskites.

### 3.5 Photoluminescence Quantum Yield in the Presence/Absence of CTAB

To quantitatively evaluate the emission evolution, the PLQY is determined based on eq 1 and it always takes values between 0 and 1.^31^
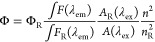
1where Φ is the PLQY, ∫*F*(λ_em_) is the integrated intensity of emission, *A*(λ_ex_) is the percentage of light absorbed at the excitation wavelength, *n* is the refractive index, and the subscript R denotes the reference data. The reference used to determine the photoluminescence quantum yield was quinine sulfate dihydrate having Φ = 0.546 and an emission range of 400–600 nm. The synthesized CsPbBr_3_ was analyzed using the fluorometery technique at 200 and 40 °C in the absence of CTAB. According to the obtained data, for both temperatures, the highest quantum yield was obtained when cesium oleate was added after 40 min, meaning that the formation of lead bromide perovskites is higher when the reaction mixture is heated for a longer time. As proven earlier, this is due to the fact that within time, maximum quantities of lead bromide molecules are being combined to OAm and OA, inducing the enhancement of the CsPbBr_3_ yield.

Moreover, decreasing the temperature from 200 to 40 °C has shown a remarkable effect on the PLQY values. In fact, according to Thomson et al., the % of PLQY decreases nonlinearly as the temperature increases, where the value of the PLQY increased from 0.02 at 200 K and reached a maximum of 0.43 at about 80 K.^32^ A similar pattern of PLQY values was obtained in our case when decreasing the temperature to 40 °C. Hence, upon lowering the temperature from 200 to 40 °C, the PLQY increases from 0.037 to 0.167 for *t* = 40 min. Therefore, at 40 °C, the PLQY of CsPbBr_3_ increased remarkably over 4 times, higher than the values obtained at 200 °C (see Table SI.2). This increase in the PLQY while decreasing the temperature was proven to be due to the immobility of the charge carriers at low temperatures and thus their inability to reach the nonradiative recombination centers. As temperature decreases, the immobile charge carriers will combine radiatively and thus increase the PLQY.^32,34^ Moreover, the effect of CTAB was established on the PLQY values. Interestingly, the PLQY % of CsPbBr_3_ was enhanced to reach 0.75 when CTAB was added to the solution. It is remarkable that the presence of CTAB boosts the PLQY ∼4-fold. This increase is due to the enhancement of the stability of the synthesized perovskites upon the addition of CTAB, which is mainly related to the presence of methylammonium groups. Different PLQY values found in the literature are shown in Table SI.3.

## 4 Conclusions

In conclusion, CsPbBr_3_ was synthesized through a simple method by the hot injection process. It was found that the most stable and smallest CsPbBr_3_ NPs were formed when 1.2 mL of cesium oleate was added in the presence of 0.15 g of PbBr_2_ heated for 40 min. The formed NPs were obtained with 3D structures with moderate stability. Furthermore, it was verified that doping with CTAB was critical in terms of the increase of the photoluminescence stability and thermal stability and in boosting the PLQY. Hence, the addition of CTAB has enhanced the stability of the PL intensity peak of the formed CsPbBr_3_, where the PL intensity decreases by only ∼15% within 4 days. Furthermore, the presence of CTAB has stabilized the incorporation of PbBr_2_ inside the perovskites, where the formed CsPbBr_3_ loses only around ∼10% of its total mass. Finally, the PLQY was boosted from 0.167 to 0.75 in the presence of CTAB.

## Supporting Information Available

Emission maximum wavelength/peaks, photoluminescence quantum yield, SEM imgaes, XRD, and TGA of lead bromide perovskites (PDF)
