# Supplementary material for: Cesium Lead Bromide Perovskites: Synthesis, Stability, and Photoluminescence Quantum Yield Enhancement by Hexadecyltrimethylammonium Bromide Doping
Source: ACS Omega. 2022 Jun 7;7(24):20872–80. doi: 10.1021/acsomega.2c01490 (PMC9219059; doi:10.1021/acsomega.2c01490)
Supplement: Supplementary file 1 — ao2c01490_si_001.pdf [file ao2c01490_si_001.pdf]

Supporting information

Cesium Lead Bromide Perovskites: Synthesis, Stability and Photoluminescence Quantum Yield Enhancement by Hexadecyltrimethylammonium Bromide Doping

*Christina Al Tawil, Riham El Kurdi, Digambara Patra**
Department of Chemistry, American University of Beirut, Beirut, Lebanon
Email: dp03@aub.edu.lb

Table S1: Emission wavelength of $PbBr_2$ and $CsPbBr_3$ at different time.

Time (minutes)	λ_1 (nm)	λ_2 (nm)
**0**	472	518
**10**	472	518
**20**	472	504
**40**	472	500

Table S2: PLQY of $CsPbBr_3$ at different time and temperature.

	PLQY	PLQY
**Time (min)**	**40 °C**	**200 °C**
**0**	0.1024	0.0038
**10**	0.1176	0.0056
**20**	0.1307	0.019
**40**	0.167	0.037

Table S3: different PLQY values obtained in the literature

Perovskites prepared	PLQY
CTAB-mediated antisolvent vapor route to shalelike C_s4PbBr_6 microplates[33]	0.59
CsPbBr3 Nanocrystal Solid-State Films[34]	0.54
Bismuth-Doped Hybrid Lead Bromide Perovskite Nanocrystals[35]	0.64
methylammonium lead bromide/formate mixture[36]	0.69
CTAB dopped $CsPbBr_3$ (our work)	0.75

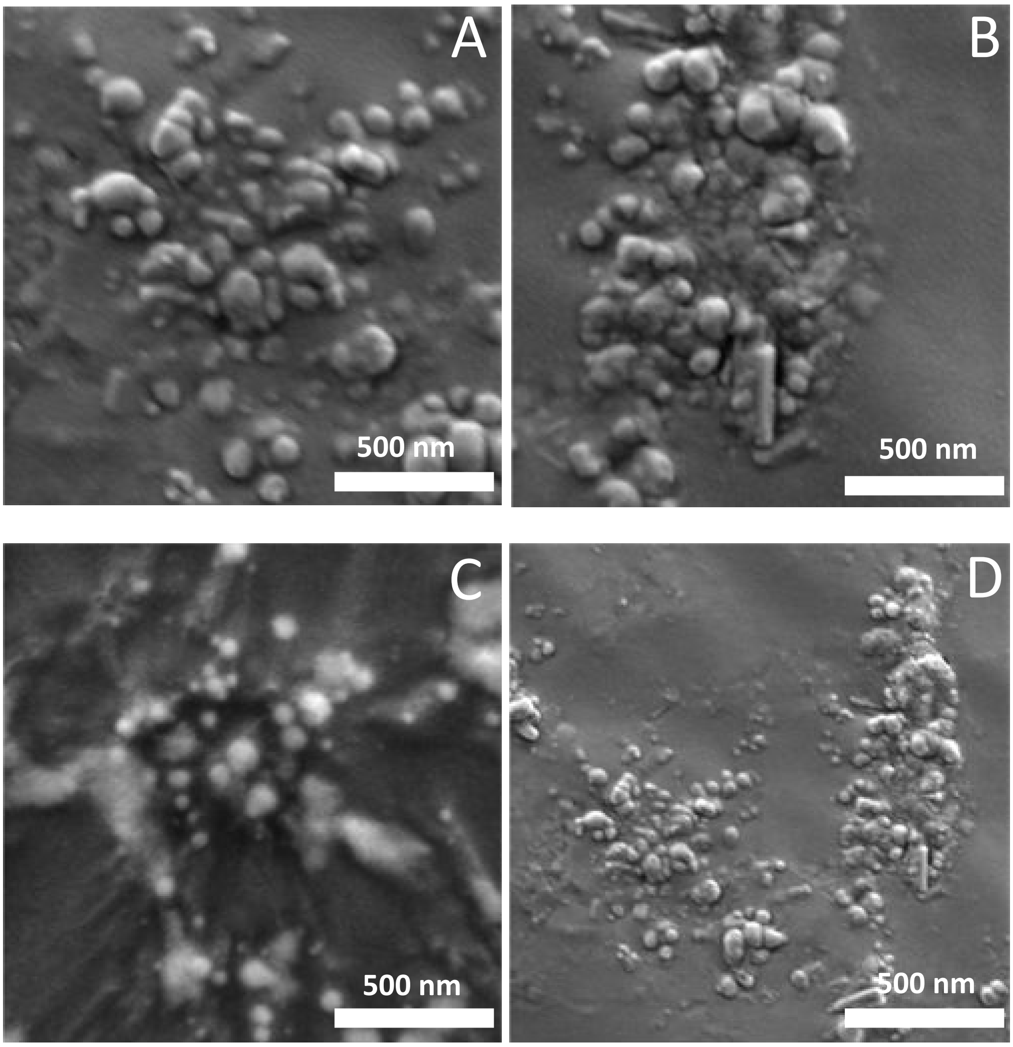

Figure S1: SEM images of lead bromide perovskites at (A) 0 min; (B) 10 min(C) 20 min; (D) 40 min.

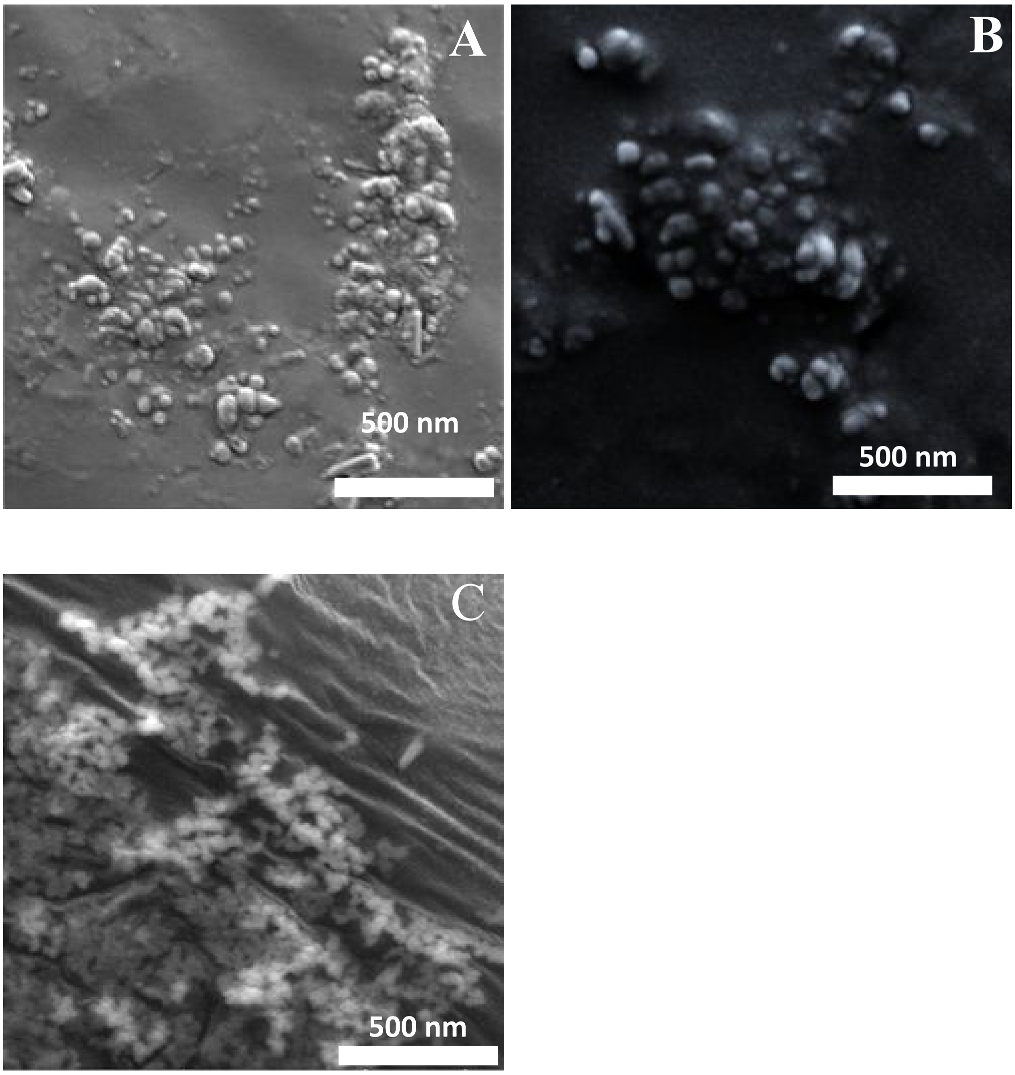

Figure S2: SEM images of lead bromide perovskites with different volumes of cesium oleate (A) 0.4 mL; (B) 0.8 mL; (C) 1.2 mL after 40 minutes.

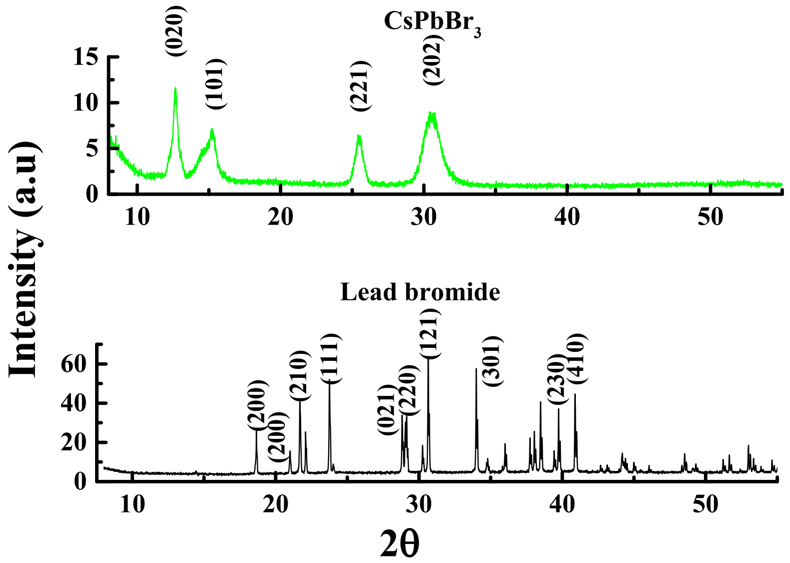

Figure S3: X-Ray Diffractogram pattern of $CsPbBr_3$ prepared using the optimized method and lead bromide precursor.

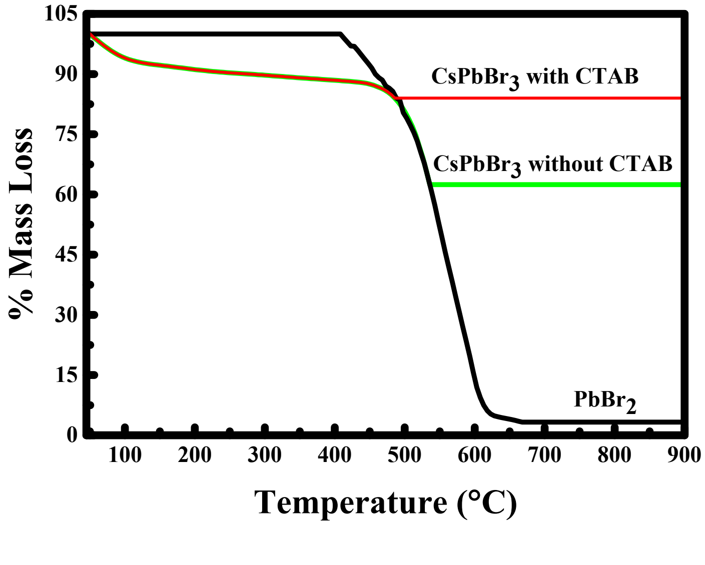

Figure S4: Thermogravimetric analysis TGA of lead bromide perovskites.
